# High-flow nasal cannula improves clinical efficacy of airway management in patients undergoing awake craniotomy

**DOI:** 10.1186/s12871-020-01073-z

**Published:** 2020-06-27

**Authors:** Ping Yi, Qiong Li, Zhoujing Yang, Li Cao, Xiaobing Hu, Huahua Gu

**Affiliations:** 1 Department of Anesthesiology, Huashan Hospital, Fudan University, No.12 Wulumuqi Zhong Road, Shanghai, 200040 China; 2 Department of Anesthesiology, Shanghai Jiahui International Hospital, Shanghai, 200000 China

**Keywords:** Awake craniotomy, High-flow nasal cannula (HFNC); nasopharyngeal airway (NPA), Gastric antral volume, Adverse events, Intracranial pressure

## Abstract

**Background:**

Awake craniotomy requires specific sedation procedure in an awake patient who should be able to cooperate during the intraoperative neurological assessment. Currently, limited number of literatures on the application of high-flow nasal cannula (HFNC) in the anesthetic management for awake craniotomy has been reported. Hence, we carried out a prospective study to assess the safety and efficacy of humidified high-flow nasal cannula (HFNC) airway management in the patients undergoing awake craniotomy.

**Methods:**

Sixty-five patients who underwent awake craniotomy were randomly assigned to use HFNC with oxygen flow rate at 40 L/min or 60 L/min, or nasopharynx airway (NPA) device in the anesthetic management. Data regarding airway management, intraoperative blood gas analysis, intracranial pressure, gastric antral volume, and adverse events were collected and analyzed.

**Results:**

Patients using HFNC with oxygen flow rate at 40 or 60 L/min presented less airway obstruction and injuries. Patients with HFNC 60 L/min maintained longer awake time than the patients with NPA. While the intraoperative PaO_2_ and SPO_2_ were not significantly different between the HFNC and NPA groups, HFNC patients achieved higher PaO_2_/FiO_2_ than patients with NPA. There were no differences in Brain Relaxation Score and gastric antral volume among the three groups as well as before and after operation in any of the three groups.

**Conclusion:**

HFNC was safe and effective for the patients during awake craniotomy.

**Trial registration:**

Chinese Clinical Trial Registry, CHiCTR1800016621. Date of Registration: 12 June 2018.

## Background

Awake craniotomy is commonly performed for resection of epileptic lesions or tumors located close to or into the functionally essential motor, cognitive, or sensory cortical areas [1]. It allows continuous monitoring of patients’ neurological functions throughout the surgery to minimize iatrogenic language or motor deficits. However, this technique brings challenges both to the neurosurgeon and anesthesiologist. The anesthetic management for this type of surgery must include sedation, analgesia, respiratory and hemodynamic control, and a responsive, co-operative patient for neurologic testing intra-operatively. There is a growing trend of preference for awake craniotomy as the approach for the removal of tumors in the sensitive cortical area has been established over the last few decades.

Airway management in the anesthesia for awake craniotomy is always concerned by anesthesiologists. Up to date, a series of venting devices including nasal cannula [2], simple facemask [3], bilateral nasopharyngeal [4], laryngeal mask [5], and endotracheal tube [6] have been used in the awake craniotomy. When these methods were applied, the patient’s head is fixed during the surgical procedure, and potential laryngospasm or cough occur when the patient is awake, which may result in surgical bleeding, increased intracranial pressure or neurological injury. Thus, endotracheal intubation or laryngeal mask, and a deeper grade of sedation/anesthesia (BIS value at 40–60) are required for the patients to prevent coughing and laryngospasm. Consequently, it takes a longer time for the patient to recover from anesthesia. Furthermore, it is difficult to re-establish the airway when the patient is inducted into the state of being asleep again [6–8]. The spontaneous breathing can be maintained under mild to moderate sedation (BIS value 60–80) through nasopharynx or oropharyngeal airways. However, nasopharyngeal or oropharyngeal airways could not completely relieve upper airway obstruction, and concentration of inhaled oxygen cannot be adjusted. In addition, nasopharyngeal airway may cause injury to nasopharynx, and the airway may be obstructed by secretions or blood clot. Furthermore, some patients may have difficulty in tolerating the nasopharyngeal or oropharyngeal airways or feel uncomfortable due to the dry airway.

Currently, while there is still no consensus or any established protocol for the best airway management for awake craniotomy, in recent years, a novel oxygen supply device, a high-flow nasal cannula (HFNC), has been introduced into medical practice [9–11]. HFNC is capable of delivering humidified (100% humidity) and heated (37 °C) oxygen at a maximum flow rate of 60 L/min [11, 12]. HFNC has presented many potential advantages over traditional oxygen supply devices, including decreased nasopharyngeal resistance, washing out of the nasopharyngeal dead space, generation of positive pressure in the pharynx, increasing alveolar recruitment in the lungs, humidification of the airways, increased fraction of inspired oxygen and improved mucociliary clearance [13–18]. Emerging evidence indicates that HFNC is effective in various clinical settings, such as acute respiratory failure [11, 19, 20], acute heart failure [21, 22], postoperative hypoxemia after cardiac surgery [23, 24], during sedation and analgesia [25]. However, there is no published study on the investigation of clinical efficacy and safety of HFNC in patients undergoing awake craniotomy. Therefore, we designed this study to evaluate the clinical outcomes of HFNC by comparing HFNC with NPA in the anesthesia management for awake craniotomy. The primary endpoint of this study was to determine if HFNC could be safely used during awake craniotomy, and secondary endpoint was to determine if HFNC is superior to the traditional NPA in terms of outcomes and safety in the awake craniotomy.

## Methods

### Study population

We collected medical data of patients who underwent awake craniotomy at our hospital from June 2018 to July 2019. This manuscript adheres to the applicable CONSORT guidelines. This clinical trial was approved by the Institutional Ethics Committee (approval number: KY2018–232) and registered at http://www.chictr.org.cn/index.aspx (registration number: CHiCTR1800016621).

The inclusion criteria: 1). Patients were 14–70 years of age. No gender preference; 2). Intracranial tumors or epileptic lesions located in the eloquent brain areas and its peripheral areas, wake-up anesthesia was required in craniotomy; 3). American Society of Anesthesiologists (ASA) physical status: Grade I or II; 4). Patients had no aphasia or changes in muscle strength before surgery.

The exclusion criteria: 1). Patients had severe organ diseases and were in decompensation (such as medical severe complications: a. Cardiac functional capacity ≥ class III; b. Respiratory failure; c. Hepatic and renal dysfunction; d. Hematological diseases; e. Uncontrolled hypertension; f. Patients with a history of COPD, pulmonary fibrosis, or long-term heavy smoking before surgery; g. Patients with severe intracranial hypertension, or even had cerebral herniation before surgery); 2). Patients who were extremely fear of surgery and were expected to have difficulty in cooperating during the operation; 3). Patients with conscious or cognitive dysfunction before surgery; 4). Patients who were unable to communicate well before surgery; 5). Patients with morbid obesity (BMI ≥ 40) accompanied by obstructive sleep apnea syndrome; 6). Patients had difficult airways; 7). Patients suffered from glioma along with other tumors outside the nervous system; 8). Pregnant women; 9). Patients involved in other clinical trials in the past three months.

Sixty-five patients were eventually enrolled in this study. They were randomly assigned into the following three groups according to the airway management during anesthesia: Group 1 (*n* = 22), patients used HFNC device with an oxygen flow rate of 40 L/min (HFNC 40); Group 2 (*n* = 20), patients used HFNC device with an oxygen flow rate of 60 L/min (HFNC 60); Group 3 (*n* = 23), patients used nasopharyngeal airway (NPA). Patients were evaluated during the pre-operative visit by the anesthesiologist and the procedure was explained in detail.

### Anesthesia management

In the operating room, the peripheral intravenous catheters were set up, and standard monitors such as electrocardiograph, pulse oximeter, and non-invasive blood pressure measurement devices were connected. Invasive blood pressure was monitored after arterial cannulation with local antiesthetic (LA) infiltration in radial or dorsalis pedis artery. Bispectral index (BIS®) monitoring (A-2000; Aspect Medical Systems, Newton, MA, USA) was connected to titrate the amount of sedatives and hypnotics. Sedative drugs were injected by a pump in the following sequence: 1). A loading dose of 0.6 μg/kg dexmedetomidine was infused within 15 min. Then, dexmedetomidine was maintained at 0.1 μg/kg/h. 2). Remifentanil Target controlled Infusion model (TCI) (Ce) was maintained at 0.5–2.0 ng/ml, which started from 0.5 μg and increased by 0.5 μg every 5–10 min till respiration frequence was at least 12 times/min. When respiration was nearly 12 times/min, TCI increased by 0.25 μg till stabilized. After the scalp nerve was blocked with 20 mL of 0.75% ropivacaine + 10 mL of 2% lidocaine + 1: 200,000 of epinephrine, propofol was infused under TCI (Ce) model at the dose of 1.0–2.0 μg/ml. Specifically, titration target of propofol was to reach BIS: 60–70, and respiration frequency: 10–20 times/min. Propofol TCI was set to 1.0–2.0 μg/mL, which started from 1.0 μg and increased by 0.5 μg every 10–15 min till BIS reached 70. If BIS decreased to 60, TCI concentration increased by 0.25 μg till stabilized. Three ways of oxygen delivery were established. 1). Group I- HFNC 40, high-flow nasal cannula (HFNC) device was used, and oxygen flow rate was set at 40 L/ min, FiO_2_ 60%, airway humidified temperature was set at 34°C; 2). Group II- HFNC 60, high-flow nasal cannula (HFNC) device was used, and oxygen flow rate was set at 60 L/ min; FiO_2_ 60%, airway humidified temperature was set at 34°C; 3). Group III-nasopharyngeal airway (NPA): nasopharynx airway device was used. The end of the nasopharyngeal airway was connected to the threaded tube of anesthesia machine. The oxygen flow rate was set at 6 L/min, FiO_2_ 60% with no humidification.

When BIS value was maintained at 60–70 and the respiratory rate was maintained at 12–20 times/min by titration of propofol and remifentanil, induction of anesthesia was considered as completed. A urinary catheter was inserted. The head of the patient was fixed with a Mayfield head clamp. The body was adjusted to a comfortable position; with the head slightly elevated in order to avoid jugular venous flow compression. Such position prevents airway occlusion when the patient was asleep. The patient was asleep during the processes of scalp incision, bone flap removal, and dura suspending. Before bone flap removal, mannitol was administrated at the dose of 1.0 g/kg for 20 min. The intracranial pressure was assessed by the surgeons five seconds after removing the bone flap using Brain Relaxation Score (BRS). Specifically, by palpating and feeling the tension of the dura mater, BRS was subjectively scored by the surgeons from 1 to 10, with 10 was the most satisfied intracranial pressure control. After the dura suspending was done, propofol infusion was stopped, and the patient was allowed to wake up spontaneously. If the patient could not be awoken in 10 min after stopping propofol infusion, dexmedetomidine infusion would be decreased or stopped. After the BIS value was maintained above 90, cortical functional mapping was achieved using NIM-ECLIPSE® System (Medtronic Xomed Inc., Jacksonville, FL, USA) with a monopolar probe, delivering stimuli with a single 1 ms pulse with a 60 Hz frequency during surgical tumor resection. Upon the requirement of surgeons, the deep sedation was induced again by the titration of propofol, dexmedetomidine, and remifentanil. BIS value at 60–70 and the respiratory rate at 12–20 times/min could be considered as the completion of re-induction of anesthesia.

### Study variables

The baseline characteristics including age, gender, body mass index (BMI) and ASA physical status was collected. The following intraoperative data were collected: 1). Blood gas analysis at 6 different time points (before induction of anesthesia; 15 min after induction of anesthesia, 15 min after the adjustment of comfortable body position, dura suspension was completed, functional mapping was being performed, 15 min after re-induction of anesthesia). 2). Vital signs (heart rate, blood pressure, SpO_2_, and respiratory rate were measured every 5 min). 3). Depth of sedation/anesthesia (BIS value and OAA/S score). 4). Brain Relaxation Score, which was assessed every 15 min. 5). The time that patients took to wake up spontaneously. 6). The total time that patients were awake. 7). Total dose of each sedative drug. 8). Total anesthesia time. 9). Gastric antral volume before and after surgery. 10). Incidence of adverse events. The gastric antral volume was evaluated by measuring the cross-sectional area (CSA) of the antrum using the ultrasound [26]. The head to sacral (CC) and anteroposterior (AP) diameter of the antrum was measured. The CSA was calculated by the formula CSA = AP x CC x π/4.

### Adverse events

Information of the following adverse events was collected. 1). The incidence of respiratory tract obstruction, which was defined as no airflow, apnea, or snoring due to partial airway obstrction. 2). Airway injury, which was defined as blood or bloody secretion found on the tube of NPA or in the patients’ mouth. 3). Increased intracranial pressure that required instant treatment.

### Statistical analysis

The sample size was calculated using PASS11 software. By ANOVA, took SPO_2_ as major parameter, that is, gave SPO_2_ as 100, 95, and 97 for HFNC 40, HFNC 60 and NPA, respectively, and a was 0.05. Sample number was from 5 to 40 with 5 as interval, and standard deviations were 2, 4, and 5. Statistical power and sample size were then calculated. When sample number was 20 and SD was 5, 0.8 of the statistical power was obtained; if SD was 2, 1 statistical power was obtained. Therefore, 20 was chosen as the sample size of each group.

The categorical variables were expressed as the frequency (%), and the Chi-square test was used for comparison. The measurable variables were expressed as mean ± SD, representation or median (interquartile range). Differences between groups were compared using One-way ANOVA when normal distribution was achieved, followed by Student-Newman-Keuls (SNK) test. If the normal distribution was not achieved, the Kruskal-Wallis test was used. Comparison within group, that is, before and after operation, was performed by Paired Student *t* test. All tests were two-tailed and statistical significance was accepted at *P* <  0.05. All statistical analysis was performed with SAS 9.2.

## Results

### Baseline characteristics

This study enrolled 65 patients who underwent awake craniotomy and supplied oxygen via HFNC or NPA. Baseline characteristics of patients were presented in Table 1. There was no significant difference in age, gender ratio, BMI, presence of epilepsy or hypertension, and types of surgery among three groups (Table 1).

**Table 1:** Baseline characteristics of the participants

Variables	Index of variables	HFNC 40 (*n* = 22)	HFNC 60 (*n* = 20)	NPA (*n* = 23)
Age (years)	Mean ± SD	37.32 ± 15.28	41.25 ± 13.89	40.43 ± 10.16
Body Mass Index (kg/m^2^)	Mean ± SD	23.71 ± 3.68	23.45 ± 4.16	21.81 ± 2.29
Gender	Male	11 (50.00)	13 (65.00)	11 (47.83)
	Female	11 (50.00)	7 (35.00)	12 (52.17)
ASA physical status	I	10 (45.45)	11 (55.00)	16 (69.57)
	II	12 (54.55)	9 (45.00)	7 (30.43)
Epilepsy	No	18 (81.82)	18 (90.00)	18 (78.26)
	Yes	4 (18.18)	2 (10.00)	5 (21.74)
Hypertension	No	20 (90.91)	19 (95.00)	23 (100.0)
	Yes	2 (9.09)	1 (5.00)	0 (0.00)
Surgery type	Other surgery	4 (18.18)	1 (5.00)	2 (8.70)
	Right-sided glioma resection	7 (31.82)	2 (10.00)	7 (30.43)
	Left-sided glioma resection	11 (50.00)	17 (85.00)	14 (60.87)

*HFNC* high-flow nasal cannula, *NPA* nasopharyngeal airway, *ASA* American Society of Anesthesiologists

### Intraoperative data of the patients using HFNC or NPA devices

#### Blood gas analysis

There were no significant differences in SpO_2_ and PaCO_2_ at various time points during surgery among HFNC 40, HFNC 60 and NPA groups (Table 2). However, patients using HFNC 40 or HFNC 60 treatment achieved higher PaO_2_/FiO_2_ than patients using the nasopharyngeal airway at various time points (HFNC 40 vs. NPA or HFNC 60 vs. NPA, all *P* <  0.05, Table 2). In addition, in this study, mild to moderate sedation generated high but acceptable PaCO_2_ level in all three groups at the end of dura suspension although the differences of PaCO_2_ before and after the anesthesia were significant in all three groups (HFNC 40: 10.80 ± 4.40; HFNC 60: 10.60 ± 3.91; NPA: 12.25 ± 5.10, *P* <  0.01, Table 3).

**Table 2:** Intraoperative blood gas analysis among three groups

Variables	Sample collection time point	HFNC 40 (*n* = 22)	HFNC 60 (*n* = 20)	NPA (*n* = 23)
SpO_2_	Before induction of anesthesia	98.2 ± 1.4	97.4 ± 2.0	97.5 ± 1.2
	15 min after induction of anesthesia	99.4 ± 1.0	99.6 ± 0.5	99.8 ± 0.4
	15 min after achieving position	99.6 ± 0.7	99.5 ± 0.6	99.7 ± 0.6
	End of dura suspension	99.6 ± 0.7	99.6 ± 0.5	99.8 ± 0.4
	Cortical functional mapping	99.5 ± 0.8	99.8 ± 0.3	99.9 ± 0.2
	15 min after re-induction	99.7 ± 0.7	99.6 ± 0.6	99.7 ± 0.5
PaCO_2_	Before induction of anesthesia	39.4 ± 3.7	38.6 ± 4.7	39.5 ± 4.9
	15 min after induction of anesthesia	46.2 ± 4.6	45.8 ± 7.3	49.6 ± 6.6
	15 min after achieving position	48.0 ± 4.3	47.9 ± 6.3	50.7 ± 6.2
	End of dura suspension	50.2 ± 4.1	49.2 ± 6.1	51.7 ± 6.2
	Cortical functional mapping	44.1 ± 2.8	42.3 ± 4.9	43.6 ± 5.9
	15 min after re-induction	47.0 ± 4.3	46.0 ± 5.0	48.3 ± 5.4
PaO_2_/FiO_2_	Before induction of anesthesia	451.8 ± 69.4	421.9 ± 112.7	447.8 ± 64.9
	15 min after induction of anesthesia	475.5 ± 81.7	496.00 ± 80.54	332.1 ± 115.0^*#^
	15 min after achieving position	500.5 ± 93.6	499.45 ± 73.21	376.9 ± 92.1^*#^
	End of dura suspension	477.6 ± 103.8	464.2 ± 90.8	384.3 ± 98.6^*#^
	Cortical functional mapping	475.0 ± 106.1	465.4 ± 78.0	275.1 ± 92.8^*#^
	15 min after re-induction	488.1 ± 100.4	494.7 ± 81.0	315.6 ± 93.9^*#^

Data were expressed as mean ± SD. *P < 0.05 compared with HFNC 40 group; ^#^*P* < 0.05 compared with HFNC 60 group. HFNC: high-flow nasal cannula; NPA: nasopharyngeal airway

**Table 3:** PaCO_2_ alteration at the end of dura suspension compared to before surgery

Group	Sample Number	Difference (Mean ± SD)	Median (Interquartile range)	*P* value
HFNC 40	22	10.80 ± 4.40	10.75 (6.50,13.90)	< 0.001^a^
HFNC 60	20	10.60 ± 3.91	11.00 (6.95,12.85)	< 0.001^a^
NPA	23	12.25 ± 5.10	12.50 (8.10,14.90)	< 0.001^a^

^a^Compared to the value before surgery. HFNC: high-flow nasal cannula

*NPA* nasopharyngeal airway

#### Brain relaxation score and gastric antral volume

There were no differences in Brain Relaxation Score at the end of the dura suspension and during the period of cortical functional mapping among the three groups (Table 4). Furthermore, no differences were noted in gastric antral volume among the three groups as well as before and after operation in any of the three groups (Table 4).

**Table 4:** Brain Relaxation Score and gastric antral volume among three groups

Group	Brain Relaxation Score		Gastric antral volume (L)	
	End of dura suspension	Functional mapping	Preoperative	Postoperative
HFNC 40 (*n* = 22)	7.9 ± 1.6	8.6 ± 0.8	1.6 ± 0.9	2.0 ± 0.4
HFNC 60 (*n* = 20)	7.3 ± 1.3	8.7 ± 2.1	1.7 ± 0.4	2.1 ± 0.4
NPA (*n* = 23)	7.0 ± 1.9	8.1 ± 1.1	1.6 ± 0.3	1.9 ± 0.4

Data were expressed as mean ± SD. There was no significant difference in any pair of comparison. L: liter; HFNC: high-flow nasal cannula; NPA: nasopharyngeal airway

#### Anesthesia duration, time that patients took to wake up and the time that patients maintained awake

There were no differences in anesthesia duration and the time that patients took to wake up spontaneously among the three groups (Table 5). However, the awake time maintained in the patients receiving HFNC 60 treatment (141.5 [98.0, 198.5]) was longer than that in the patients received HFNC 40 (105.0 [75.0, 136.0], *P* <  0.05, Table 5) or NPA treatment (99.0 [85.0, 113.0], *P* <  0.05, Table 5), respectively.

**Table 5:** Comparison of anesthesia duration, time that patients took to wake up and the time that patients maintained awake

Variables	HFNC 40 (n = 22)	HFNC 60 (n = 20)	NPA (*n* = 23)
Anesthesia Duration (min)	366.5 (300.0, 3933.0)	380 (321.5407.5)	385.0 (340.0,404.0)
Time patients took to wake up (min)	8.0 (6.0,12.0)	7.0 (6.0,11.0)	8.0 (7.0,13.0)
Awakening Duration (min)	105.0 (75.0,136.0)	141.5 (98.0,198.5) ^*^	99.0 (85.0,113.0) ^#^

Data were expressed as median (interquartile range). *P < 0.05 compared with HFNC 40; ^#^*P* < 0.05 compared with HFNC 60. HFNC: high-flow nasal cannula; NPA: nasopharyngeal airway

#### Total sedative drugs used by patients

There were no differences in the total dose of dexmidiatomidine, propofol or remifentanil used throughout the whole surgery among the three groups (*P* > 0.05, Table 6).

**Table 6:** Total sedative medications used for the patients

Medications	HFNC 40 (*n* = 22)	HFNC 60 (*n* = 20)	NPA (*n* = 23)
Dexmediatomidine (μg)	76.3 (67.6,83.2)	80.5 (59.7102.4)	82.0 (63.0,115.0)
Remifentanil (mg)	0.43 (0.35,0.48)	0.39 (0.27,0.51)	0.45 (0.36,0.56)
Propofol (mg)	403.5 (318.0,575.0)	397.25 (340.0,608.0)	442.0 (273.0,500.0)

Data were expressed as median (interquartile range). HFNC: high-flow nasal cannula; NPA: nasopharyngeal airway

#### Incidence of adverse events

The incidence of respiratory tract obstruction in NPA group was 43% (10 out of 23 patients), which was significantly higher than that in HFNC 40 (3 out of 22 patients, 13%, *P* <  0.05) or HFNC 60 group (1 out of 20 patients, 5%, *P* < 0.05, Table 7). No patient presented airway injury (blood or bloody secretion found on the tube of NPA or in the patients’ mouth) in HFNC 40 or HFNC 60 group (Table 7). However, 6 patients in the NPA group suffered from airway injury, which was significantly higher than that in the patients using HFNC (all *P* < 0.05, Table 7). Three patients in HFNC 40 group, five patients in HFNC 60 group, and six patients in NPA group presented increased Brain Relaxation Score, and appropriate treatment, including mannitol infusion, body position change (head high and feet low), or decreased dose of anesthesia drugs, was required to reduce intracranial pressure. There were no differences in the incidence of intracranial pressure enhancement among the three groups (*P* > 0.05, Table 7).

**Table 7:** Incidence of adverse events among three groups

Adverse events	Variable level	HFNC 40 (*n* = 22)	HFNC 60 (*n* = 20)	NPA (*n* = 23)
Obstruction of upper airway	No	19 (86.3)	19 (95.0)	13 (56.5)
	Yes	3 (13.6)^*^	1 (5.0)^*^	10 (43.5)
Airway injury	No	22 (100.0)	20 (100.0)	17 (73.9)
	Yes	0 (0.0)^*^	0 (0.0)^*^	6 (26.1)
Requiring treatment for increased Brain Relaxation Score	No	19 (86.4)	15 (75.0)	17 (73.9)
	Yes	3 (13.6)	5 (25.0)	6 (26.1)

*P < 0.05 compared with NPA. HFNC: high-flow nasal cannula; NPA: nasopharyngeal airway. “Obstruction of upper airway” was defined as no airflow, apnea or snoring due to partial airway obstruction. “Airway injury” was defined as blood or bloody secretion was found on the tube of NPA or in the patients’ mouth

## Discussion

To our knowledge, this was the first study evaluating the efficacy and safety of HFNC application in the anesthesia management for awake craniotomy. As compared with NPA group, HFNC 40 or HFNC 60 treatment resulted in similar physiological response including intraoperative SpO_2_ and PaCO_2_, Brain Relaxation Score at the end of dura suspension or during the period of cortical functional mapping, and the gastric antral volume before and after anesthesia. However, both HFNC 40 and HFNC 60 treatments achieved higher PaO_2_/FiO_2_ ratio than NPA did. Furthermore, neither HFNC 40 nor HFNC 60 treatment caused respiratory tract injury while NPA did cause the injury. In addition, less airway obstruction occurred in the patients given HFNC 40 or 60, and longer awake time was observed in the patients with HFNC 60.

In recent years, HFNC has become a world-wide popular strategy in clinical practice for the delivery of humidified and heated oxygen in the treatment of the critically ill patient who requires high inspiratory oxygen therapy [9]. It has been reported that humidified high flow oxygen may benefit not only mucociliary clearance and mobilization of respiratory secretions [27, 28], but also increasing patient comfort and reducing mucus injury [10, 11, 18, 23, 29, 30]. Furthermore, it does not impede mobility, oral intake, or speaking [31, 32]. Consistent with these studies, in the current study, neither HFNC 40 nor HFNC 60 treatment resulted in airway injury, while 26% of patients in NPA group presented airway injury. Furthermore, patients given HFNC 40 or 60 presented lower incidence of airway obstruction as compared with patients given NPA. This advantage of HFNC may be due to the increased nasopharyngeal pressure generated by high flow oxygen. In support of this concept, a similar phenomenon was observed in McGinley’s study [33]. They reported that high flow oxygen alleviated obstructive apnea-hypopnea syndrome in 11 patients [33]. This phenomenon could be associated with the enhanced nasopharyngeal pressure at the end of exhalation, which resulted in decreased airway subsidence and subsequently relieved respiratory obstruction [15, 34, 35].

In this study, high flow oxygen generated acceptable PaCO_2_ and desired PaO_2_. Although three patients in the HFNC 40 group presented increased PaCO_2_, it dropped to normal range when the oxygen flow was increased to 60 L/min, indicating that HFNC could generate a certain degree of continuous positive airway pressure (CPAP)-like effect, which depended on both flow rate and mouth position (open versus closed) [14]. Nevertheless, PaCO_2_ level was significantly increased in all three treatment groups at each checking time point without significant differences among the three groups, suggesting PaCO_2_ could be affected by multiple factors in addition to the oxygen flow amount, and thus, it should be closely monitored by the Anethesiologist during the process of awake craniotomy.

The intracranial pressure was assessed by surgeons subjectively and expressed as Brain Relaxation Score in this study. During the processes of dura suspending and tumor resection, Brain Relaxation Score in both HFNC groups was maintained at the level that surgeons desired to have, suggesting intracranial pressure was not significantly affected by high flow oxygen inhalation.

In this study, all patients maintained spontaneous breath throughout the surgical process. One of the adverse effects of high flow oxygen inhalation could be gastric discomfort. Therefore, gastric antral volumes before and after anesthesia were compared among the three groups. We found that gastric antral volume did not change after anesthesia in any of the study groups, suggesting that HFNC do not lead to gas accumulation in the stomach and cause gastric discomfort. Furthermore, none of the HFNC patient needed invasive airway device during the surgery. To maintain the patient’s sedation depth during the surgery, doses of anesthetics were adjusted according to the BIS value and the OAA/S score, which was satisfied by the sugeons and met the requirement of anesthesia management.

We recommended that initial flow rate be set at 40 L/min, which could be increased during the operation, if the patient have upper airway obstruction or other complications. When the upper airway obstruction cannot be relieved by increasing the inspired flow or position adjustment, airway management device (such as nasopharynx or oropharyngeal airway) must be immediately applied.

## Conclusion

The current study demonstrated that application of HFNC 40 or 60 during awake craniotomy resulted in higher ratio of PaO_2_/FiO2, longer awaken time (HFNC 60), but less airway injury or obstruction compared to that of NPA. These findings suggested that nasal high-flow oxygen inhalation device can be safely and effectively used in the anesthesia management for awake craniotomy. However, findings of the current study should be confirmed in the morbid obesity patients in the future.

## Data Availability

The datasets generated and analyzed during the present study are available from the corresponding author on reasonable request.

## Abbreviations

HFNC: High-flow nasal cannula
LA: Local antiesthetic
BMI: Body mass index
CSA: The cross-sectional area
CPAP: Continuous positive airway pressure

## References

[1] Sahjpaul RL (2000). Awake craniotomy: controversies, indications and techniques in the surgical treatment of temporal lobe epilepsy. Can J Neurol Sci.

[2] Skucas AP, Artru AA (2006). Anesthetic complications of awake craniotomies for epilepsy surgery. Anesth Analg.

[3] Picht T, Kombos T, Gramm HJ, Brock M, Suess O (2006). Multimodal protocol for awake craniotomy in language cortex tumour surgery. Acta Neurochir.

[4] Sivasankar C, Schlichter RA, Baranov D, Kofke WA (2016). Awake craniotomy: a new airway approach. Anesth Analg.

[5] Murata H, Nagaishi C, Tsuda A, Sumikawa K (2010). Laryngeal mask airway supreme for asleep-awake-asleep craniotomy. Br J Anaesth.

[6] Deras P, Moulinie G, Maldonado IL, Moritz-Gasser S, Duffau H, Bertram L (2012). Intermittent general anesthesia with controlled ventilation for asleep-awake-asleep brain surgery: a prospective series of 140 gliomas in eloquent areas. Neurosurgery.

[7] Cai T, Gao P, Shen Q, di Zhang Z, Yao Y, Ji Q (2013). Oesophageal naso-pharyngeal catheter use for airway management in patients for awake craniotomy. Br J Neurosurg.

[8] Audu PB, Loomba N (2004). Use of cuffed oropharyngeal airway (COPA) for awake intracranial surgery. J Neurosurg Anesthesiol.

[9] Zhang J, Lin L, Pan K, Zhou J, Huang X (2016). High-flow nasal cannula therapy for adult patients. J Int Med Res.

[10] Spoletini G, Alotaibi M, Blasi F, Hill NS (2015). Heated humidified high-flow nasal oxygen in adults: mechanisms of action and clinical implications. Chest.

[11] Roca O, Riera J, Torres F, Masclans JR (2010). High-flow oxygen therapy in acute respiratory failure. Respir Care.

[12] Kubicka ZJ, Limauro J, Darnall RA (2008). Heated, humidified high-flow nasal cannula therapy: yet another way to deliver continuous positive airway pressure?. Pediatrics.

[13] Sztrymf B, Messika J, Bertrand F, Hurel D, Leon R, Dreyfuss D, Ricard JD (2011). Beneficial effects of humidified high flow nasal oxygen in critical care patients: a prospective pilot study. Intensive Care Med.

[14] Groves N, Tobin A (2007). High flow nasal oxygen generates positive airway pressure in adult volunteers. Aust Crit Care.

[15] Parke R, McGuinness S, Eccleston M (2009). Nasal high-flow therapy delivers low level positive airway pressure. Br J Anaesth.

[16] Moller W, Celik G, Feng S, Bartenstein P, Meyer G, Oliver E, Schmid O, Tatkov S (2015). Nasal high flow clears anatomical dead space in upper airway models. J Appl Physiol (1985).

[17] Dewan NA, Bell CW (1994). Effect of low flow and high flow oxygen delivery on exercise tolerance and sensation of dyspnea. A study comparing the transtracheal catheter and nasal prongs. Chest.

[18] Dysart K, Miller TL, Wolfson MR, Shaffer TH (2009). Research in high flow therapy: mechanisms of action. Respir Med.

[19] Diaz-Lobato S, Folgado MA, Chapa A, Mayoralas Alises S (2013). Efficacy of high-flow oxygen by nasal cannula with active humidification in a patient with acute respiratory failure of neuromuscular origin. Respir Care.

[20] Rochwerg B, Granton D, Wang DX, Helviz Y, Einav S, Frat JP, Mekontso-Dessap A, Schreiber A, Azoulay E, Mercat A (2019). High flow nasal cannula compared with conventional oxygen therapy for acute hypoxemic respiratory failure: a systematic review and meta-analysis. Intensive Care Med.

[21] Kang MG, Kim K, Ju S, Park HW, Lee SJ, Koh JS, Hwang SJ, Hwang JY, Bae JS, Ahn JH (2019). Clinical efficacy of high-flow oxygen therapy through nasal cannula in patients with acute heart failure. J Thorac Dis.

[22] Carratala Perales JM, Llorens P, Brouzet B, Albert Jimenez AR, Fernandez-Canadas JM, Carbajosa Dalmau J, Martinez Beloqui E, Ramos Forner S (2011). High-flow therapy via nasal cannula in acute heart failure. Rev Esp Cardiol.

[23] Parke RL, McGuinness SP, Dixon R, Jull A (2012). Protocol for a randomised controlled trial of nasal high flow oxygen therapy compared to standard care in patients following cardiac surgery: the HOT-AS study. Int J Nurs Stud.

[24] Nicolet J, Poulard F, Baneton D, Rigal JC, Blanloeil Y (2011). High-flow nasal oxygen for severe hypoxemia after cardiac surgery. Ann Fr Anesth Reanim.

[25] Deitch K, Chudnofsky CR, Dominici P, Latta D, Salamanca Y (2011). The utility of high-flow oxygen during emergency department procedural sedation and analgesia with propofol: a randomized, controlled trial. Ann Emerg Med.

[26] Cubillos J, Tse C, Chan VW, Perlas A (2012). Bedside ultrasound assessment of gastric content: an observational study. Can J Anaesth.

[27] Chanques G, Constantin JM, Sauter M, Jung B, Sebbane M, Verzilli D, Lefrant JY, Jaber S (2009). Discomfort associated with underhumidified high-flow oxygen therapy in critically ill patients. Intensive Care Med.

[28] Hasani A, Chapman TH, McCool D, Smith RE, Dilworth JP, Agnew JE (2008). Domiciliary humidification improves lung mucociliary clearance in patients with bronchiectasis. Chron Respir Dis.

[29] Ward JJ (2013). High-flow oxygen administration by nasal cannula for adult and perinatal patients. Respir Care.

[30] Maggiore SM, Idone FA, Vaschetto R, Festa R, Cataldo A, Antonicelli F, Montini L, De Gaetano A, Navalesi P, Antonelli M (2014). Nasal high-flow versus Venturi mask oxygen therapy after extubation. Effects on oxygenation, comfort, and clinical outcome. Am J Respir Crit Care Med.

[31] Peters SG, Holets SR, Gay PC (2013). High-flow nasal cannula therapy in do-not-intubate patients with hypoxemic respiratory distress. Respir Care.

[32] Roca O, Hernandez G, Diaz-Lobato S, Carratala JM, Gutierrez RM, Masclans JR (2016). Spanish multidisciplinary Group of High Flow Supportive Therapy in a: current evidence for the effectiveness of heated and humidified high flow nasal cannula supportive therapy in adult patients with respiratory failure. Crit Care.

[33] McGinley BM, Patil SP, Kirkness JP, Smith PL, Schwartz AR, Schneider H (2007). A nasal cannula can be used to treat obstructive sleep apnea. Am J Respir Crit Care Med.

[34] Badiee Z, Eshghi A, Mohammadizadeh M (2015). High flow nasal cannula as a method for rapid weaning from nasal continuous positive airway pressure. Int J Prev Med.

[35] Jeong JH, Kim DH, Kim SC, Kang C, Lee SH, Kang TS, Lee SB, Jung SM, Kim DS (2015). Changes in arterial blood gases after use of high-flow nasal cannula therapy in the ED. Am J Emerg Med.

